# Supplementary material for: Social connections are differentially related to subjective age and physiological age acceleration amongst older adults
Source: Nat Commun. 2026 Jan 30;17:2173. doi: 10.1038/s41467-026-68977-1 (PMC12960813; doi:10.1038/s41467-026-68977-1)
Supplement: Supplementary file 2 — Reporting Summary [file 41467_2026_68977_MOESM2_ESM.pdf]

nature portfolio

Corresponding author(s): Daisy Fancourt

Last updated by author(s): Jan 21, 2025

Reporting Summary

Nature Portfolio wishes to improve the reproducibility of the work that we publish. This form provides structure for consistency and transparency in reporting. For further information on Nature Portfolio policies, see our Editorial Policies and the Editorial Policy Checklist.

Statistics

For all statistical analyses, confirm that the following items are present in the figure legend, table legend, main text, or Methods section.

n/a	Confirmed	
☐	☒	The exact sample size (*n*) for each experimental group/condition, given as a discrete number and unit of measurement
☐	☒	A statement on whether measurements were taken from distinct samples or whether the same sample was measured repeatedly
☐	☒	The statistical test(s) used AND whether they are one- or two-sided<br>*Only common tests should be described solely by name; describe more complex techniques in the Methods section.*
☐	☒	A description of all covariates tested
☐	☒	A description of any assumptions or corrections, such as tests of normality and adjustment for multiple comparisons
☐	☒	A full description of the statistical parameters including central tendency (e.g. means) or other basic estimates (e.g. regression coefficient) AND variation (e.g. standard deviation) or associated estimates of uncertainty (e.g. confidence intervals)
☐	☒	For null hypothesis testing, the test statistic (e.g. *F*, *t*, *r*) with confidence intervals, effect sizes, degrees of freedom and *P* value noted<br>*Give P values as exact values whenever suitable.*
☒	☐	For Bayesian analysis, information on the choice of priors and Markov chain Monte Carlo settings
☒	☐	For hierarchical and complex designs, identification of the appropriate level for tests and full reporting of outcomes
☐	☒	Estimates of effect sizes (e.g. Cohen's *d*, Pearson's *r*), indicating how they were calculated

Our web collection on statistics for biologists contains articles on many of the points above.

Software and code

Policy information about availability of computer code

Data collection	No software was used for data collection
Data analysis	Full code is available here: https://github.com/dfancourt/socialdeficits

For manuscripts utilizing custom algorithms or software that are central to the research but not yet described in published literature, software must be made available to editors and reviewers. We strongly encourage code deposition in a community repository (e.g. GitHub). See the Nature Portfolio guidelines for submitting code & software for further information.

Data

Policy information about availability of data

All manuscripts must include a data availability statement. This statement should provide the following information, where applicable:

- Accession codes, unique identifiers, or web links for publicly available datasets
- A description of any restrictions on data availability
- For clinical datasets or third party data, please ensure that the statement adheres to our policy

ELSA data are publicly available here https://beta.ukdataservice.ac.uk/datacatalogue/series/series?id=200011#!/abstract

Research involving human participants, their data, or biological material

Policy information about studies with human participants or human data. See also policy information about sex, gender (identity/presentation), and sexual orientation and race, ethnicity and racism.

Reporting on sex and gender	Only sex is collected within ELSA. Disaggregated data are provided with exact sample sizes.
Reporting on race, ethnicity, or other socially relevant groupings	Factors identified as predicting both receptive arts engagement and mortality were identified using directed acyclic graphs (DAGs) and included as covariates. Demographic and socio-economic confounders included chronological age (in years), gender (male or female), ethnicity (white British vs other), educational qualifications (no educational qualifications; education to GCE/O-levels/national vocational qualification (NVQ) 2 (qualifications at age 16); education to NVQ3/GCE/A-levels (qualifications at age 18); higher qualification/NVQ4/NVQ5/degree), total non-pension wealth (which combines net financial and physical wealth plus net owner-occupied housing wealth; categorised in quintiles), and house ownership (whether individuals owned their property outright vs with a mortgage/renting/social housing/other). Data were analysed using doubly robust estimation using the inverse-probability-weighted regression adjustment (IPWRA) estimator. This involves building two models to account for the non-random treatment assignment: a regression adjustment model for the outcome and a treatment-assignment model for the exposure, only one of which has to be correctly specified, enhancing the robustness of the analysis.
Population characteristics	Of the 7,047 adults included in the analyses, the average age was 65.6 years (SD 9.3) and 55.2% were female. Full population characteristics are provided in Table 1 of the manuscript.
Recruitment	Data were derived from the English Longitudinal Study of Ageing (ELSA). ELSA is a large-scale panel study of people aged 50 and over and their partners, living in private households in England. The original sample was drawn from participants from the Health Survey in England (HSE) in 1998, 1999 and 2001.
Ethics oversight	ELSA received ethical approval from the National Health Service Research Ethics Service.

Note that full information on the approval of the study protocol must also be provided in the manuscript.

Field-specific reporting

Please select the one below that is the best fit for your research. If you are not sure, read the appropriate sections before making your selection.

☐ Life sciences ☒ Behavioural & social sciences ☐ Ecological, evolutionary & environmental sciences

For a reference copy of the document with all sections, see nature.com/documents/nr-reporting-summary-flat.pdf

Behavioural & social sciences study design

All studies must disclose on these points even when the disclosure is negative.

Study description	Quantitative secondary data analysis of cohort study
Research sample	Representative sample of adults over the age of 50 living in England
Sampling strategy	Random. Data were derived from the English Longitudinal Study of Ageing (ELSA). ELSA is a large-scale panel study of people aged 50 and over and their partners, living in private households in England. The original sample was drawn from participants from the Health Survey in England (HSE) in 1998, 1999 and 2001.
Data collection	Data were collected by nurses during an interview and home visit, with additional questions responded to in a postal questionnaire.
Timing	The first wave of data collection commenced in 2002/2003, and participants have been followed biennially since. We used wave 2 (2004/5), as this wave included a rich battery of social and physiological variables of relevance.
Data exclusions	We restricted participants to core ELSA members who had returned the self-completion questionnaire where most of our main variables of interest were measured (n=8,354), who were over the age of 50 (n=8,129), and who had full data on exposures and confounders (n=7,047). Within this sample, 6,621 had data on perceived ageing, and 4,169 had full data on physiological ageing. In main analyses, the final sample was allowed to vary between analyses for perceived and physiological age to maximise power, but sensitivity analyses restricted the sample just to individuals who had both perceived and physiological ageing (n=3,928).
Non-participation	The total sample size we could have analysed was 8,780. So non-participation was 426 (<5%)
Randomization	N/A

Reporting for specific materials, systems and methods

We require information from authors about some types of materials, experimental systems and methods used in many studies. Here, indicate whether each material, system or method listed is relevant to your study. If you are not sure if a list item applies to your research, read the appropriate section before selecting a response.

Materials & experimental systems

n/a	Involved in the study
☒	☐ Antibodies
☒	☐ Eukaryotic cell lines
☒	☐ Palaeontology and archaeology
☒	☐ Animals and other organisms
☒	☐ Clinical data
☒	☐ Dual use research of concern
☒	☐ Plants

Methods

n/a	Involved in the study
☒	☐ ChIP-seq
☒	☐ Flow cytometry
☒	☐ MRI-based neuroimaging

Plants

Seed stocks	N/A
Novel plant genotypes	N/A
Authentication	N/A
